# Autistic Traits Are Not a Strong Predictor of Binocular Rivalry Dynamics

**DOI:** 10.3389/fnins.2018.00338

**Published:** 2018-05-17

**Authors:** Katie M. Wykes, Laila Hugrass, David P. Crewther

**Affiliations:** Centre for Human Psychopharmacology, Faculty of Health, Arts and Design, Swinburne University of Technology, Melbourne, VIC, Australia

**Keywords:** binocular rivalry, autistic tendency, excitation/inhibition imbalance, perception, vision, psychophysics

## Abstract

It has been suggested that differences in binocular rivalry switching rates and mixed percept durations in ASD could serve as a biomarker of excitation/inhibition imbalances in the autistic brain. If so, one would expect these differences to extend to neurotypical groups with high vs. low levels of autistic tendency. Previous studies did not detect any correlations between binocular rivalry dynamics and Autism Spectrum Quotient (AQ) scores in neurotypical control groups; however it is unclear whether this was due to the characteristics of the rivalry stimuli that were used. We further investigated this possibility in a sample of neurotypical young adults. The binocular rivalry stimuli were simple gratings, complex objects, or scrambled objects, which were presented dichoptically, either at fixation or in the periphery. A Bayesian correlation analysis showed that individuals with higher AQ scores tended to have lower perceptual switching rates for the centrally presented, simple grating rival stimuli. However, there was no evidence of a relationship between AQ and switching rates, reversal rates or mixed percept durations for any of the other binocular rivalry conditions. Overall, our findings suggest that in the non-clinical population, autistic personality traits are not a strong predictor of binocular rivalry dynamics.

## Introduction

Autism spectrum disorder (ASD) is diagnosed based on social and behavioral abnormalities (American Psychiatric Association, 2013). Recently, sensory differences have become part of the clinical description, with particular emphasis on differences in visual perception (Dakin and Frith, 2005; Simmons et al., 2009; Robertson and Baron-Cohen, 2017). These visual differences extend to the neurotypical population, for individuals with low (AQ < 12) and high (AQ > 23) levels of autistic personality traits (Jackson et al., 2013; Stevenson et al., 2016), as measured with the Autism Spectrum Quotient (AQ) (Baron-Cohen et al., 2001). Several theories for the neural basis of ASD have been proposed, one of which suggests there is an excitation/inhibition (E/I) imbalance that may extend through all cortical systems (Rubenstein and Merzenich, 2003; Vattikuti and Chow, 2010). It has been proposed that binocular rivalry could be a useful behavioral tool to investigate the E/I imbalance in autism (Robertson et al., 2013, 2016; Said et al., 2013; Freyberg et al., 2015).

Binocular rivalry occurs when conflicting images are presented to each eye, resulting in alternations between the two perceptual states over time (Wheatstone, 1838; Blake and Logothetis, 2002). It is known that larger stimuli produce higher levels of mixed perception (Blake et al., 1992) and that simple, grating stimuli elicit weaker binocular rivalry and higher levels of mixed perception than complex, object stimuli (Alais and Melcher, 2007). Although the timing of binocular rivalry is stochastic, perceptual switching rates are relatively stable within individuals and highly variable between individuals (Aafjes et al., 1966). Interestingly, binocular rivalry rates are slower in bipolar disorder (Miller et al., 2003) and twin studies suggest that the rate of perceptual switching is strongly influenced by genetics (Miller et al., 2010).

Models of binocular rivalry dynamics involve reciprocal excitatory (i.e., glutamate) and inhibitory (i.e., GABA) connections, and noise (Laing and Chow, 2002; Wilson, 2003; Noest et al., 2007; Kang et al., 2010). These models predict that either increased cortical glutamate or GABA would decrease the mean duration of mixed percepts, and speed perceptual switching rates (Said et al., 2013). Consistent with this prediction, in the neurotypical population, higher GABA concentrations are associated with decreased durations of mixed perception (Robertson et al., 2016). Yet, contrary to this prediction it has been demonstrated that higher GABA concentration in the visual cortex is associated with slower perceptual switching in binocular rivalry, and other forms of bistable perception such as motion induced blindness and ambiguous structure from motion (van Loon et al., 2013).

Robertson et al. (2013) compared binocular rivalry dynamics in groups of high functioning ASD (DSM IV definition) and healthy control participants (with no differences in non-verbal IQ scores between the two groups). Relative to the healthy control group, the ASD group tended to have slower perceptual switching rates, higher rates of reversions (transitions from a dominant percept to a mixed percept and then back to the original percept), and increased durations of mixed perception. A control experiment with yoked replays of binocular rivalry showed that these effects are unlikely to reflect differences in perceptual reporting characteristics between the two groups. These results have been replicated by Robertson et al. (2016), who also reported that the strong correlation between GABA metabolites and mixed percept durations in healthy controls is not present in ASD. Contrary to Robertson et al. (2013) and Said et al. (2013) did not find any differences between groups of ASD and control participants in mixed percept durations or the time it takes for waves of perceptual dominance to travel across rival stimuli.

One possible explanation for these discrepancies could be that Said et al. (2013) studied binocular rivalry between small grating stimuli (approximately 1°), whereas the other researchers (Robertson et al., 2013, 2016) used larger (approximately 2.7°), more complex stimuli such as a baseball bat and a piece of broccoli. To investigate this possibility, Freyberg et al. (2015) compared binocular rivalry in ASD and control groups, using both complex objects (2.8° by 2.3°) and simple gray-scale gratings (3.5° by 3.5°). Consistent with Robertson et al. (2013), perceptual transitions were slowed and mixed percept durations were lengthened in the ASD group. This effect was larger for simple stimuli than complex stimuli; however this may reflect the odd shapes of the complex stimuli, such that competitive interactions would have differed greatly in strength across the overlapping and non-overlapping regions of the two images. Also, given that complex stimuli differ from simple gratings both in terms of low-level features (i.e., complex, broadband spatial frequencies) and high-level features (object category), it is unclear whether the effects of stimulus complexity on rivalry in ASD should be interpreted in terms of feedback from object-processing regions of visual cortex, or local competitive interactions within V1.

Aside from the E/I theory, there are other differences in visual processing that may help to explain differences in binocular rivalry for ASD and control groups. For instance, although people with ASD are capable of perceiving global form, their attention tends to be biased toward local image features (Plaisted et al., 1998; Simmons et al., 2009). This local processing bias extends to the neurotypical population, for groups with high levels of autistic personality traits (Grinter et al., 2009; Stewart et al., 2009; Jackson et al., 2013; Crewther and Crewther, 2014; Stevenson et al., 2016; DiCriscio and Troiani, 2018). For example, in a study comparing groups of neurotypical participants with high (*M* = 24.4) and low (*M* = 7.7) AQ scores, perception of a bistable local/global image tended to be biased toward the local interpretation in the high AQ group, and this effect was greater in the periphery than at the fovea (Crewther and Crewther, 2014). The effects of stimulus complexity on the strength of binocular rivalry suggest that global feedback from ventral object processing regions organizes local competitive interactions in the primary visual cortex (Alais and Melcher, 2007). Hence, a local processing bias might contribute to the reduced binocular rivalry strength in ASD (i.e., longer mixed percepts and slower switching).

Given that visual processing abnormalities in ASD often extend to neurotypical individuals with high levels of autistic personality traits (Jackson et al., 2013), it seems odd that Robertson et al. (2013) and Freyberg et al. (2015) reported weak or non-significant correlations between AQ scores and binocular rivalry dynamics in their control groups. These experiments did not include a peripheral binocular rivalry condition, so it is worthwhile reassessing whether the differences in binocular rivalry dynamics in ASD can be extended to non-clinical groups with varying levels of autistic personality traits. Here we aimed to investigate the relationship between AQ scores and binocular rivalry dynamics for a neurotypical population, using both simple gratings and complex objects that were presented either centrally or in the periphery. To account for differences in the low-level features of simple and complex images, we also included a condition with scrambled objects as the binocular rivalry stimuli.

Overall, we predicted there would be moderate correlations between AQ scores and rivalry dynamics, with switching rates tending to be slower, reversion rates tending to be higher and mixed percept durations tending to be longer for those with higher AQ scores. Based on results from Freyberg et al. (2015), it was expected that these effects would be greater for simple rivalry stimuli than for complex rivalry stimuli. Furthermore, based previous results (Crewther and Crewther, 2014), it was predicted the relationship between AQ scores and rivalry dynamics would be stronger for peripherally presented stimuli than for centrally presented stimuli. In order to test for individual differences in response latencies or reporting strategies, we also included a “replay” condition in which observers were presented with physically alternating stimuli of random duration matching the cadence of binocular rivalry alternations. Based on the existing literature (Robertson et al., 2013), it was predicted that individual differences in response characteristics would not explain the relationship between AQ scores and binocular rivalry dynamics.

## Methods and materials

### Participants

Written informed consent was obtained from all participants in accordance with the protocol approved by Swinburne University of Technology Human Research Ethics Committee and in accordance with the code of ethics of the Declaration of Helsinki. The study was advertised online to university students and staff. Participants who completed the online AQ and demographics scale were invited to attend a laboratory session if they had normal or corrected-to-normal vision, and no history of neurological or psychiatric conditions, such as epilepsy or attention-deficit-hyperactivity disorder.

Forty six adults with AQ scores ranging from 4 to 42 (11 males, *M* = 18.05 years, *SD* = 9.25 years) participated in the experiment. In total, three participants were excluded from the analysis, one was color-blind, one did not experience binocular rivalry and one misinterpreted the key-press instructions. Two participants were taking antidepressant medication and one participant was taking antianxiety medication; however removing these participants from the analysis did not qualitatively change the results.

### Autism spectrum quotient

All participants completed an online version of the AQ (Baron-Cohen et al., 2001). The AQ is a scale of 50 questions related to social and environmental lifestyle choices. Participants answered questions such as “I prefer to do things with others rather than on my own” or “I would rather go to a library than to a party” on a 4-point Likert scale (definitely agree to definitely disagree).

### Stimuli and apparatus

**Figure 1:**
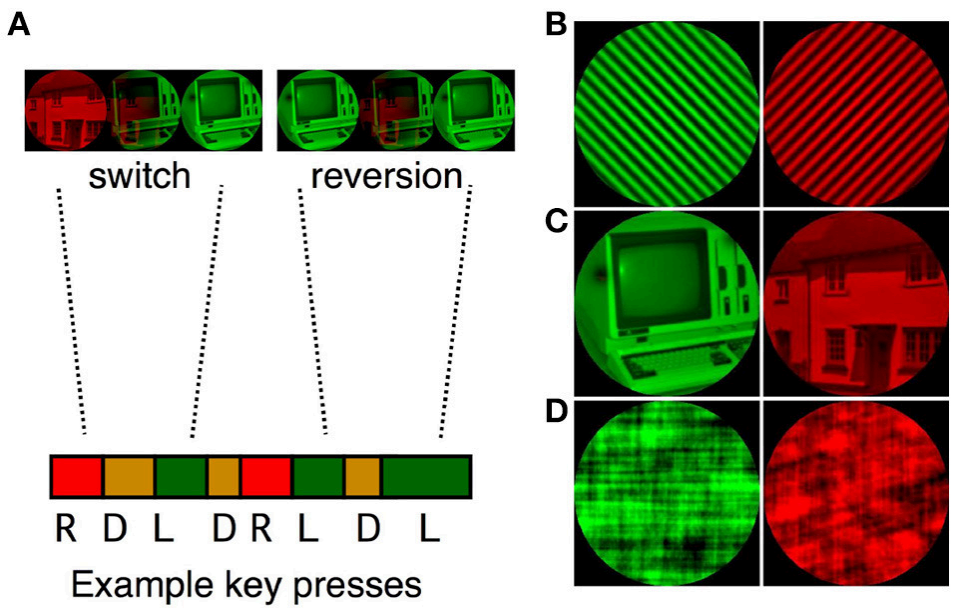
**(A)** A sample sequence of binocular rivalry transitions. Participants were instructed to report red, mixed and green percepts by pressing the keyboard right, down and left arrows respectively. Transitions were classified as switches (e.g., R, D, L) or reversions (e.g., R, D, R). Examples of the simple grating **(B)**, complex object **(C)**, and scrambled object **(D)** pairs are presented on the right of the figure.

We created sets of three binocular rivalry stimuli for each of the stimulus complexity levels (see Figure 1 for examples of simple, complex and scrambled images). The set of simple stimuli included two pairs of orthogonally oriented gratings (2.5 and 3.5 cpd), and one pair of radial/concentric gratings (4 and 16 cycles respectively). The set of object stimuli was made up of three images of houses paired with three images of computers. The scrambled stimuli were created in Matlab by phase-scrambling the object stimuli. A one-pixel blur was added to remove sharp edges from each of the images (using Photoshop, Adobe Systems). A Matlab script was used to match the images for mean luminance and RMS contrast. All images were displayed within a 4.5° circular mask, on a black background (L = 1.60 cd/m^2^, CIE_x_ = 0.33, CIE_y_ = 0.39). To improve reporting of perceptual dominance and mixed perception, the images were tinted red (L = 80 cd/m^2^, CIE_x_ = 0.68, CIE_y_ = 0.33) and green (perceptually matched to the red luminance, CIE_x_ = 0.11, CIE_y_ = 0.78).

All binocular rivalry tasks were programmed using VPixx software (version 3.20, VPixx.com) and presented using a PROPixx projector (refresh rate = 240 Hz, or 120 Hz to each eye), with linearized color output (as measured with a ColorCal colorimeter). The images were rear projected onto a polarization-preserving screen through a Depth-Q circular polarizer. The participant was seated at a viewing distance of 57 cm from the screen, and wore passive polarized lenses so that one image was visible through each eye (except in the “replay” condition, where the images were visible through both eyes). To account for individual differences in red and green perception, participants adjusted the luminance of a green scrambled image to that of a red scrambled image until both were seen as equally bright, and had roughly equal perceptual dominance durations. Despite our attempts to match the colors for perceptual brightness, repeated measures *t*-test comparisons revealed that dominance durations tended to be longer for the green tinted stimuli than for the red tinted stimuli across all binocular rivalry conditions (*p* < 0.05). Given that the effects of color and dominant eye were counterbalanced, it is unlikely that differences in color strength affected our results.

In order to test for individual differences in perceptual reporting criteria, sets of “replay” rivalry stimuli were created. Perceptual transitions were mimicked by physically alternating the images. Periods of “mixed dominance” and “perceptual reversions” were seeded from Gaussian patches that expanded or contracted across the images. “Dominance durations” for the red and green images were varied randomly (*M* = 2.05 s, *SD* = 1.5 s), as were the transition durations (*M* = 1.08 s). To replicate real binocular rivalry, either two or three reversions were included in each of the 60 s runs of the replay condition.

### Procedure

Participants were instructed to indicate whether they perceived the red image, the green image, or a mixture of the two images by continuously pressing the right, left and down keyboard arrows respectively. Participants completed a 3 min practice task, with 30 s runs of the six conditions (3 complexities × 2 eccentricities). This was repeated until they felt comfortable using the keyboard arrows to report their perceptual transitions.

For the binocular rivalry experiment, observers completed 36 × 63 s trials. The first 3 s of key-press data from each trial were discarded, so we recorded approximately 6 min of binocular rivalry for each condition. In order to counterbalance the effects of eye dominance and stimulus color, each stimulus pair was presented twice. To avoid fatigue, the experiment was split into four blocks of nine trials. Participants were encouraged to take self-timed breaks in between trials and longer breaks (>5 min) in between the blocks of conditions. For the replay task, the stimuli were presented using the same display parameters and task instructions as in the main experiment.

### Data analysis

The keyboard presses were sampled approximately every 4 ms. Custom scripts were written in Labview (National Instruments) to transform the key-press data into continuous time sequences of perceptual state (red dominant, green dominant or mixed). The first 3 s of each trial were omitted from the analysis. Periods when no key was pressed and any key-press durations shorter than 150 ms were marked as “mixed.” For each experimental condition, percept duration data from the six trials were concatenated in order to calculate the median duration for each percept (green, red, and mixed), and rate of perceptual switches and reversions. Switches were defined as sequences when perception shifted from one state to another (red to green dominance, with or without an intermediate mixed percept). Reversions were defined as sequences when perception reverted to the previously reported color following a mixed percept (red-mixed-red or green-mixed-green). Outliers and bivariate outliers (i.e., unusual combination of scores on two variables) were identified and removed from the dataset prior to statistical analysis.

Statistical analyses were performed using JASP (Version 0.8.4, JASP Team, 2018). Kendall's Tau correlations were performed to test the relationships between AQ scores and switching rates, reversion rates and mixed percept durations. Bayes factors allow researchers to compare the level of evidence for a tested hypothesis against the null hypothesis. A *BF*_10_ of 10 or *BF*_01_ of 0.1 would indicate that the data are 10 times more likely to be explained in favor of H_1_ than H_0_. As a rule of thumb, Bayes factors greater than three or less than 0.3 are considered as evidence for or against a hypothesis, and Bayes factors between 0.3 and 3 are not considered worth mentioning. Bayes factors for the correlations are based on the analysis technique proposed by Wetzels and Wagenmakers (2012).

## Results

### Descriptive statistics

We have presented sample means and standard deviations for binocular rivalry dynamics (i.e., switch rates, reversal rates and median mixed durations) in Table 1, for each of the stimulation conditions. The full dataset is available as Supplementary Material. Repeated measures ANOVAs were conducted to investigate the effects of stimulus condition on binocular rivalry dynamics. As illustrated in Table 1, there was a significant effect of stimulus complexity on switch rates for both the central [*F*_(2, 84)_ = 76.45, *p* < 0.001, η_*p*_^2^ = 0.65] and peripheral conditions [*F*_(2, 84)_ = 17.61, *p* < 0.001, η_*p*_^2^ = 0.30]. For both the central and peripheral viewing conditions, perceptual switching rates tended to be faster for simple than complex (central; *t* = 5.77, *p* < 0.001, peripheral; *t* = 2.31 *p* = 0.023) or scrambled stimuli (central; *t* = 6.59, *p* < 0.001, peripheral; *t* = 3.58, *p* < 0.001). For perceptual reversals, there was a significant effect of stimulus type for the peripheral condition [*F*_(2, 84)_ = 6.15, *p* < 0.003, η_*p*_^2^ = 0.13], but not for the central presentation condition [*F*_(2, 84)_ = 2.85, *p* < 0.135]. For the peripheral stimuli, reversals tended to occur less frequently for the complex stimuli than for the simple stimuli (*t* = 2.61, *p* = 0.011). For the central presentation condition there was a significant effect of stimulus complexity on mixed percept durations [*F*_(2, 74)_ = 16.90, *p* < 0.001, η_*p*_^2^ = 0.32], on averaged participants tended to have lower median mixed percept durations for the simple gratings than for the complex images (*t* = 3.53, *p* < 0.001). For the peripheral condition, there was no effect of stimulus type on mixed percept durations [*F*_(2, 80)_ = 1.93, *p* = 0.152].

**Table 1:** Descriptive statistics for switching rates, reversal rates, and median mixed durations.

	**Switch rate (min**^**−1**^**)**			**Reversal rate (min**^**−1**^**)**			**Mixed duration (s)**		
**Stimulus**	***M***	***SD***	***N***	***M***	***SD***	***N***	***M***	***SD***	***N***
**CENTRAL**									
Simple	21.12	6.30	43	7.68	4.98	41	1.49	1.15	42
Complex	14.88	4.32	43	5.88	3.60	43	2.15	1.61	41
Scramble	14.64	4.92	43	7.08	4.86	43	1.91	1.08	40
**PERIPHERAL**									
Simple	18.78	6.72	43	9.96	5.94	43	2.01	1.56	42
Complex	15.78	5.10	43	7.56	5.16	43	1.94	1.25	43
Scramble	15.30	5.82	43	8.34	4.56	43	2.29	1.48	42

### Switching rate

**Figure 2:**
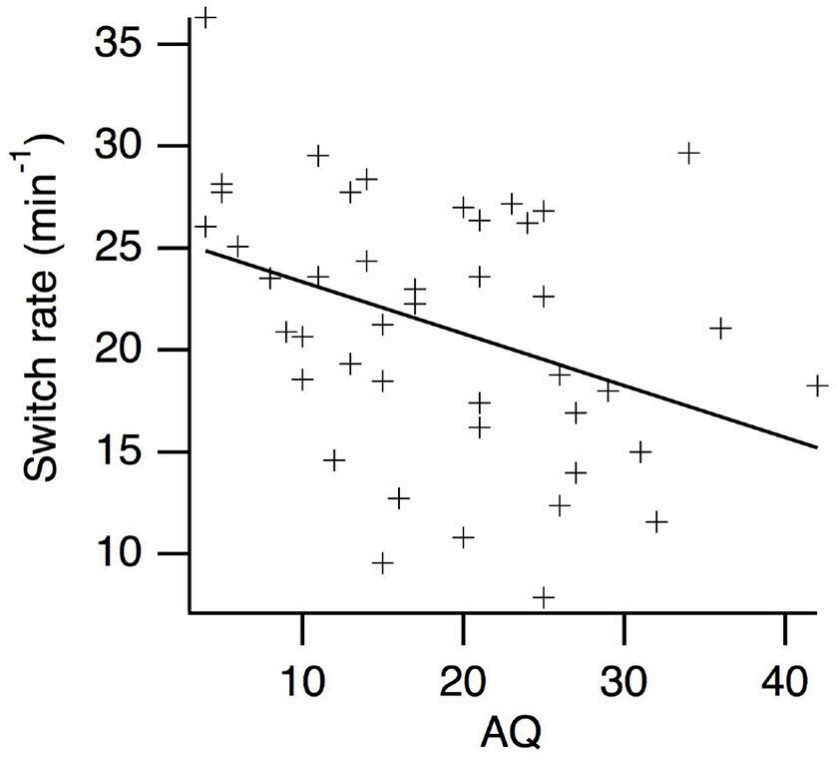
Scatter plot of perceptual switching rates vs. AQ scores for the centrally presented, simple grating rival stimuli.

Kendall's Tau correlations between AQ scores and perceptual switching rates are presented in Table 2. As expected, there was a weak, negative relationship between AQ scores and perceptual switching rates for the centrally presented, simple grating stimuli (see Figure 2). The BF_10_ indicates that evidence for a correlation is 5.90 times stronger than evidence against a correlation. Contrary to expectation, there were no substantial correlations between switching rates and AQ scores for any of the other measures. For central scrambled (*BF*_01_ = 4.39), peripheral complex *(BF*_01_ = 4.41) and peripheral scrambled (*BF*_01_ = 3.05) rivalry conditions, the observed data are more than three times more likely to be explained by the null hypothesis than by a correlation with AQ scores. By contrast, there were moderate, positive correlations between participants' perceptual switching rates for the different binocular rivalry conditions, with strong Bayesian evidence for these correlations. Hence, although individual variations in switching rates tend to be consistent across different measures of binocular rivalry, AQ scores are not highly predictive of this variation.

**Table 2:** Kendall's Tau correlations for AQ scores and perceptual switching rates.

		**Central**			**Peripheral**		
	**AQ**	**Simple**	**Complex**	**Scram**	**Simple**	**Complex**	**Scram**
AQ	—						
Central simple	−0.28^†^	–					
Central complex	−0.12	0.62^†††^	–				
Central scram	−0.06	0.45^†††^	0.55^†††^	–			
Periphery simple	−0.14	0.35 ^††^	0.42^†††^	0.34 ^††^	–		
Periphery complex	−0.06	0.44^†††^	0.53^†††^	0.42^†††^	0.57^†††^	–	
Periphery scram	−0.11	0.42 ^††^	0.46^†††^	0.40^†††^	0.56^†††^	0.66^†††^	–

† *BF_10_ > 5*,

†† *BF_10_ > 30*,

††† *BF_10_ > 100*.

### Reversion rates

Correlations between perceptual reversion rates and AQ scores are presented in Table 3. Contrary to expectation, there were no substantial correlations between AQ scores and perceptual reversion rates for any of the stimulus conditions. The Bayesian analyses supported the null-hypothesis for each of the centrally presented rivalry conditions (simple: *BF*_01_ = 4.33, complex: *BF*_01_ = 4.28, scrambled: *BF*_01_ = 4.86), and for the peripherally presented, complex (*BF*_01_ = 4.55) and scrambled (BF_01_ = 5.02) rivalry conditions. For the peripherally presented, simple gratings (*BF*_01_ = 1.81), there was no strong evidence for or against the null hypothesis. As expected, there were moderate, positive correlations between the reversion rates for the different binocular rivalry conditions, with the Bayesian analyses indicating strong evidence in support of these inter-correlations.

**Table 3:** Kendall's Tau correlations for AQ scores and perceptual reversion rates.

		**Central**			**Peripheral**		
	**AQ**	**Simple**	**Complex**	**Scram**	**Simple**	**Complex**	**Scram**
AQ	–						
Central simple	0.06	–					
Central complex	0.06	0.33^†^	–				
Central scram	0.03	0.53^†††^	0.36^††^	–			
Periphery simple	0.15	0.43^†††^	0.46^†††^	0.52^††^	–		
Periphery complex	−0.05	0.42^†††^	0.37^††^	0.50^†††^	0.47^†††^	–	
Periphery scram	−0.01	0.35^†^	0.40^†††^	0.59^†††^	0.45^†††^	0.61^†††^	–

† *BF_10_ > 5*,

†† *BF_10_ > 30*,

††† *BF_10_ > 100*.

### Mixed percept durations

Kendall's Tau correlations between mixed percept durations and AQ scores are presented in Table 4. Contrary to expectation, there were no substantial correlations between AQ and mixed percept durations for any of the stimulus conditions. The Bayesian analysis supports the null hypothesis for the complex, peripherally presented rivalry condition (*BF*_01_ = 4.01). For the other stimulus conditions, there was no strong evidence for or against the null hypothesis. A larger sample would be required in order to test these correlations. Moderate strength, positive, correlations were observed between the mixed percept durations for the different binocular rivalry conditions, although evidence for the correlation between the peripheral complex and peripheral scrambled mixed durations was not particularly strong (*BF*_10_ = 3.55).

**Table 4:** Kendall's Tau correlations for AQ scores and mixed percept durations.

		**Central**			**Peripheral**		
	**AQ**	**Simple**	**Complex**	**Scram**	**Simple**	**Complex**	**Scram**
AQ	—						
Central simple	0.12	—					
Central complex	0.20	0.66^†††^	—				
Central scram	0.16	0.50^†††^	0.59^†††^	—			
Periphery simple	0.20	0.46^†††^	0.46^†††^	0.38 ^††^	—		
Periphery complex	−0.07	0.50^†††^	0.41^†††^	0.45^†††^	0.41^†††^	—	
Periphery scram	0.12	0.47	0.45	0.41	0.54	0.26	—

† *BF_10_ > 5*,

†† *BF_10_ > 30*,

††† *BF_10_ > 100*.

### Replay condition

A “replay” experiment was conducted to investigate relationships between AQ scores and perceptual reporting criteria. Four response characteristics were computed: reaction times to report mixed images (RT_mix_), reaction times to report perceptual dominance after the end of a mixed period (RT_dom_), the percentage of switches reported accurately (% Switch), and the percentage of reversions reported accurately (% Rev). Kendall's Tau correlations between these criteria and AQ scores for the centrally and peripherally presented replay stimuli are presented in Table 5.

**Table 5:** Kendall's Tau correlations for AQ scores and “replay” response characteristics.

		**Central**				**Peripheral**			
	**AQ**	**RT_mix_**	**RT_dom_**	**%Switch**	**%Rev**	**RT_mix_**	**RT_dom_**	**%Switch**	**%Rev**
AQ	—								
**CENTRAL**									
RT_mix_	0.11	—							
RT_dom_	0.18	0.28	—						
%Switch	0.15	0.35^††^	0.15^†^	—					
%Rev	0.16	0.54^†††^	0.39^†††^	0.38^†††^	—				
**PERIPHERAL**									
RT_mix_	0.11	0.62^†††^	0.32^†^	0.34^††^	0.54^†††^	—			
RT_dom_	0.08	0.21	0.57^†††^	0.03	0.22	0.11	—		
%Switch	0.06	0.2	0.13	0.19	0.36^††^	0.22	0.04	—	
%Rev	0.21	0.55^†††^	0.44^†††^	0.36^††^	0.46^†††^	0.57^†††^	0.32^††^	0.11	—

† *BF_10_ > 5*,

†† *BF_10_ > 30*,

††† *BF_10_ > 100*.

There were no substantial correlations between “replay” response characteristics and AQ scores. The strongest AQ correlation was with the percentage of reversions reported in the peripheral condition. This may indicate that people with high AQ scores tend not to notice reversions in the periphery; however there was insufficient evidence for or against this correlation (*BF*10 = 1.38*)*. As illustrated in Table 5, there were some inter-correlations between the different reporting characteristics. This indicates that individual differences in binocular rivalry dynamics could in part reflect differences in the ways that switches are reported. However, our “replay” results suggest that the relationship we observed between AQ and binocular rivalry switching rates (for centrally presented grating stimuli) is unlikely to reflect differences in perceptual reporting criteria across the autistic personality spectrum.

## Discussion

We investigated the relationship between autistic personality traits and binocular rivalry dynamics in a sample of healthy, young adults. We were interested in whether previous studies had failed to detect a relationship between AQ scores and binocular rivalry dynamics because of their odd choice of complex rivalry stimuli or because of their lack of a peripheral binocular rivalry condition (Robertson et al., 2013; Freyberg et al., 2015). We addressed these issues by matching the low-level visual properties (i.e., RMS contrast and size) of our simple, complex, and scrambled binocular rivalry stimuli, and by including a peripheral binocular rivalry condition. Consistent with the previous studies, we did not find any significant correlations between AQ scores and binocular rivalry dynamics, with the exception of a weak relationship between AQ and perceptual switching rates for the centrally presented grating stimuli. Hence, despite previously reported differences in binocular rivalry dynamics for clinical ASD and control groups (Robertson et al., 2013, 2016; Freyberg et al., 2015), our results indicate that in the neurotypical population, autistic personality traits do not explain a substantial amount of the individual variation in binocular rivalry dynamics.

Our finding that the relationship between autistic personality and perceptual switching rates is weaker for rivalry between complex objects than simple patterns (of the same retinal size) is consistent with the results of Freyberg et al.'s (2015) study comparing ASD and control groups. The competitive interactions underlying binocular rivalry appear to increase in strength at successive levels of the visual processing hierarchy (Blake and Logothetis, 2002), such that binocular rivalry between objects tends to be more stable and coherent than rivalry between gratings (Alais and Melcher, 2007). Yet, contrary to expectation, on average, mixed percept durations were longer for complex objects than for simple grating stimuli. Although we took care to match the complex object stimuli for luminance and RMS contrast, perhaps local contrast differences between regions of the images limited the proportion of exclusive perceptual dominance. Hence, the question remains open as to whether the differences in local/global object perception for people with varying degrees of autistic personality traits (Grinter et al., 2009) contribute to the observed differences in binocular rivalry dynamics.

We found a relationship between binocular rivalry dynamics and autistic personality traits for centrally presented, but not peripherally presented grating stimuli. This was unexpected based on the results of Crewther and Crewther (2014), who found stronger effects of autistic tendency on perceptual rivalry between an ambiguous local/global diamond stimulus when it was presented in the periphery. A possible explanation for this discrepancy may be that despite commonalities in temporal dynamics (Carter and Pettigrew, 2003), different forms of perceptual rivalry might not necessarily be driven by common oscillators and inhibitory mechanisms (Jaworska and Lages, 2014). Hence, low-level receptive field sizes and attentional mechanisms may contribute differently to the effects of eccentricity on binocular rivalry and other forms of perceptual rivalry. Future studies may seek to address the ways in which individual differences in receptive field sizes, local/global processing biases, and attentional mechanisms contribute to individual differences in the dynamics of various forms of perceptual rivalries.

The absence of a clear relationship between AQ scores and binocular rivalry dynamics is surprising, given the differences in binocular rivalry dynamics for clinical ASD and control groups (Robertson et al., 2013, 2016; Freyberg et al., 2015). The AQ has been well-validated as a measure of autistic personality traits in ASD in the general population (Baron-Cohen et al., 2001). Many studies have demonstrated that visual processing differences in autism can be extended to the neurotypical population for groups with high and low AQ scores (e.g., Van Heer and Crewther, 2012; Jackson et al., 2013; Flevaris and Murray, 2015). On the contrary, our findings suggest that individual differences in the brain mechanisms underlying binocular rivalry dynamics and autistic personality traits do not overlap substantially. In the paragraphs below, we discuss several possible explanations as to why binocular rivalry differs with ASD diagnosis, but not with trait AQ.

It could be that differences in medication or co-morbid mood disorders contribute to differences in binocular rivalry dynamics for ASD and control groups. Psychotropic medications are frequently used to treat psychological and behavioral issues in adults and adolescents with ASD (Lake et al., 2015). Eleven of the 20 ASD participants in Robertson et al.'s (2016) study were on medication, and five of the 26 ASD participants in Freyberg et al.'s (2015) study were on medication. These medications ranged from antidepressants, antianxiety and antipsychotic drugs. In our non-clinical sample, two participants with high AQ scores were on antidepressant medication and one participant with a mid AQ score was on antianxiety medication. However, both in our study and in the previous clinical studies, similar effects were observed when the medicated participants were removed from the sample.

Co-morbidity may have also been an issue. Although previous studies of binocular rivalry in ASD tended to exclude participants with comorbid ADHD (Robertson et al., 2013, 2016; Freyberg et al., 2015), it is possible that other comorbid disorders influenced the results. For instance, in a study of adolescents and young adults with high functioning ASD, 36% of the sample were diagnosed with a mood disorder, and bipolar disorder accounted for 75% of these cases (Munesue et al., 2008). Binocular rivalry rates are slowed in bipolar disorder (Pettigrew and Miller, 1998; Miller et al., 2003), hence it is possible that a common bipolar/ASD phenotype underlies the differences in binocular rivalry dynamics between ASD and control groups. However, given that amine neurotransmitters are implicated in bipolar disorder, this explanation would not be consistent with Robertson et al.'s (2016) E/I explanation.

Consistent with previous studies (Aafjes et al., 1966), we found that individual differences in binocular rivalry dynamics tend to be stable across different stimulus conditions. Given the strong genetic contribution to switching rates (Miller et al., 2010), it is interesting to speculate on the neural bases of individual differences in binocular rivalry dynamics. In support of the theory that E/I ratios influence binocular rivalry dynamics (Robertson et al., 2013; Said et al., 2013), van Loon et al. (2013) found that individuals with higher GABA concentrations tend to have lower perceptual switching rates for binocular rivalry and other forms of perceptual rivalry. Interestingly, Robertson et al. (2016) found that GABA levels are correlated with the proportion of mixed perception in healthy controls, but not in ASD. This suggests that for those with ASD, individual differences in binocular rivalry dynamics are not related to their E/I ratios. Aside from the E/I ratio, there are other neural mechanisms that may explain individual differences in binocular rivalry dynamics. For example, it has been shown that individual differences in the structure of the superior parietal lobes can predict inter-individual variability in binocular rivalry rates (Kanai et al., 2010).

The small sample size is a potential limitation of the current study. Our analyses may not have been sensitive to weak relationships between AQ scores and binocular rivalry dynamics. An advantage of reporting Bayesian correlations is that it allows for quantification of evidence for the null hypothesis. For many of the correlations reported, the Bayes factors suggest there is substantial evidence against a relationship between AQ scores and binocular rivalry dynamics. For other stimulus conditions, our sample was not sufficiently large to be certain whether or not a correlation exists at the population level. However, the sample size was sufficient to detect moderate to strong relationships between binocular rivalry dynamics across the different stimulus conditions, our results suggest that AQ scores are unlikely to explain a substantial percentage of the individual variation in binocular rivalry dynamics.

In summary, we investigated the relationship between autistic personality traits and binocular rivalry dynamics in healthy young adults. Our results indicate that for the most part, there are no substantial relationships between autistic personality traits and binocular rivalry dynamics, with the exception of a weak correlation between AQ and switching rates for the centrally presented grating stimuli. The effects of stimulus complexity on perceptual switching rates were greater for people with low levels of autistic personality traits. Taken together, our findings suggest that although there are visual processing differences across the autistic personality spectrum, these differences do not explain much of the individual variation in binocular rivalry dynamics. If there is an E/I imbalance in ASD, we propose that other paradigms, such as orientation selective surround suppression (Van Heer and Crewther, 2012; Flevaris and Murray, 2015) might be more sensitive to its effects on visual processing.

## Author contributions

KW contributed to this manuscript, including creating the initial experimental design, participant testing sessions, manuscript editing, and the preparation of the manuscript. LH contributed to this manuscript by finalizing the experiment design, creating stimuli, programming analysis, creating manuscript figures, and manuscript editing. DC contributed by supervising experiment design and manuscript editing.

### Conflict of interest statement

The authors declare that the research was conducted in the absence of any commercial or financial relationships that could be construed as a potential conflict of interest.

## References

[B1] Aafjes M. Hueting J. E. Visser P. (1966). Individual and interindividual differences in binocular retinal rivalry in man. Psychophysiology 3, 18–22. 10.1111/j.1469-8986.1966.tb02674.x 5942867

[B2] Alais D. Melcher D. (2007). Strength and coherence of binocular rivalry depends on shared stimulus complexity. Vis. Res. 47, 269–279. 10.1016/j.visres.2006.09.003 17049579

[B3] American Psychiatric Association (2013). Neurodevelopmental Disorders Diagnostic and Statistical Manual of Mental Disorders, 5th Edn. Washington, DC: American Psychiatric Association.

[B4] Baron-Cohen S. Wheelwright S. Skinner R. Martin J. Clubley E. (2001). The autism-spectrum quotient (AQ): evidence from asperger syndrome/high-functioning autism, malesand females, scientists and mathematicians. J. Autism Dev. Disord. 31, 5–17. 10.1023/A:1005653411471 11439754

[B5] Blake R. Logothetis N. K. (2002). Visual competition. Nat. Rev. Neurosci. 3, 13–21. 10.1038/nrn701 11823801

[B6] Blake R. O'Shea R. P. Mueller T. J. (1992). Spatial zones of binocular rivalry in central and peripheral vision. Vis. Neurosci. 8, 469–478. 10.1017/S0952523800004971 1586647

[B7] Carter O. L. Pettigrew J. D. (2003). A common oscillator for perceptual rivalries? Perception 32, 295–305. 10.1068/p3472 12729381

[B8] Crewther D. P. Crewther D. P. (2014). Peripheral global neglect in high vs. low autistic tendency. Front. Psychol. 5:284. 10.3389/fpsyg.2014.00284 24772100 PMC3983523

[B9] Dakin S. Frith U. (2005). Vagaries of visual perception in autism. Neuron 48, 497–507. 10.1016/j.neuron.2005.10.018 16269366

[B10] DiCriscio A. S. Troiani V. (2018). The Broader autism phenotype and visual perception in children. J. Autism Dev. Disord. 48, 1–12. 10.1007/s10803-018-3534-9 29574583

[B11] Flevaris A. V. Murray S. O. (2015). Orientation-specific surround suppression in the primary visual cortex varies as a function of autistic tendency. Front. Hum. Neurosci. 8:1017. 10.3389/fnhum.2014.01017 25610385 PMC4285125

[B12] Freyberg J. Robertson C. E. Baron-Cohen S. (2015). Reduced perceptual exclusivity during object and grating rivalry in autism. J. Vis. 15, 11–11. 10.1167/15.13.11 26382002 PMC4594764

[B13] Grinter E. J. Maybery M. T. Van Beek P. L. Pellicano E. Badcock J. C. Badcock D. R. (2009). Global visual processing and self-rated autistic-like traits. J. Autism Dev. Disord. 39, 1278–1290. 10.1007/s10803-009-0740-5 19381793

[B14] Jackson B. L. Blackwood E. M. Blum J. Carruthers S. P. Nemorin S. Pryor B. A.. (2013). Magno- and parvocellular contrast responses in varying degrees of autistic trait. PLoS ONE 8:e66797. 10.1371/journal.pone.0066797 23824955 PMC3688931

[B15] JASP Team (2018). JASP (Version 0.8.6) [Computer Software].

[B16] Jaworska K. Lages M. (2014). Fluctuations of visual awareness: combining motion-induced blindness with binocular rivalry. J. Vis. 14, 11–11. 10.1167/14.11.11 25240063 PMC4168770

[B17] Kanai R. Bahrami B. Rees G. (2010). Human parietal cortex structure predicts individual differences in perceptual rivalry. Curr. Biol. 20, 1626–1630. 10.1016/j.cub.2010.07.027 20727757 PMC2949566

[B18] Kang M.-S. Lee S.-H. Kim J. Heeger D. Blake R. (2010). Modulation of spatiotemporal dynamics of binocular rivalry by collinear facilitation and pattern-dependent adaptation. J. Vis. 10:3. 10.1167/10.11.3 20884498 PMC2951267

[B19] Laing C. R. Chow C. C. (2002). A spiking neuron model for binocular rivalry. J. Comput. Neurosci. 12, 39–53. 10.1023/A:1014942129705 11932559

[B20] Lake J. K. Vogan V. Sawyer A. Weiss J. A. Lunsky Y. (2015). Psychotropic medication use among adolescents and young adults with an autism spectrum disorder: parent views about medication use and healthcare services. J. Child Adolesc. Psychopharmacol. 25, 260–268. 10.1089/cap.2014.0106 25803636

[B21] Miller S. M. Gynther B. Heslop K. Liu G. Mitchell P. Ngo T.. (2003). Slow binocular rivalry in bipolar disorder. Psychol. Med. 33, 683–692. 10.1017/S0033291703007475 12785470

[B22] Miller S. M. Hansell N. K. Ngo T. T. Liu G. B. Pettigrew J. D. Martin N. G.. (2010). Genetic contribution to individual variation in binocular rivalry rate. Proc. Natl. Acad. Sci. U.S.A. 107, 2664–2668. 10.1073/pnas.0912149107 20133779 PMC2823875

[B23] Munesue T. Ono Y. Mutoh K. Shimoda K. Nakatani H. Kikuchi M. (2008). High prevalence of bipolar disorder comorbidity in adolescents and young adults with high-functioning autism spectrum disorder: a preliminary study of 44 outpatients. J. Affect. Disord. 111, 170–175. 10.1016/j.jad.2008.02.015 18378000

[B24] Noest A. Van Ee R. Nijs M. Van Wezel R. (2007). Percept-choice sequences driven by interrupted ambiguous stimuli: a low-level neural model. J. Vis. 7:10. 10.1167/7.8.10 17685817

[B25] Pettigrew J. D. Miller S. M. (1998). A ‘sticky’interhemispheric switch in bipolar disorder? Proceed. Royal Soc. Lond. B Biol. Sci. 265, 2141–2148. 9872002 10.1098/rspb.1998.0551 PMC1689515

[B26] Plaisted K. O'Riordan M. Baron-Cohen S. (1998). Enhanced visual search for a conjunctive target in autism: a research note. J. Child Psychol. Psychiatry 39, 777–783. 10.1017/S0021963098002613 9690940

[B27] Robertson C. E. Baron-Cohen S. (2017). Sensory perception in autism. Nat. Rev. Neurosci. 18, 671–684. 10.1038/nrn.2017.112 28951611

[B28] Robertson C. E. Kravitz D. J. Freyberg J. Baron-Cohen S. Baker C. I. (2013). Slower rate of binocular rivalry in autism. J. Neurosci. 33, 16983–16991. 10.1523/JNEUROSCI.0448-13.2013 24155303 PMC3807027

[B29] Robertson C. E. Ratai E.-M. Kanwisher N. (2016). Reduced GABAergic action in the autistic brain. Curr. Biol. 26, 80–85. 10.1016/j.cub.2015.11.019 26711497

[B30] Rubenstein J. L. R. Merzenich M. M. (2003). Review model of autism: increased ratio of excitation/inhibition in key neural systems. Genes Brain Behav. 2, 255–267. 10.1034/j.1601-183X.2003.00037.x 14606691 PMC6748642

[B31] Said C. P. Egan R. D. Minshew N. J. Behrmann M. Heeger D. J. (2013). Normal binocular rivalry in autism: implications for the excitation/inhibition imbalance hypothesis. Vis. Res. 77, 59–66. 10.1016/j.visres.2012.11.002 23200868 PMC3538943

[B32] Simmons D. R. Robertson A. E. McKay L. S. Toal E. McAleer P. Pollick F. E. (2009). Vision in autism spectrum disorders. Vis. Res. 49, 2705–2739. 10.1016/j.visres.2009.08.005 19682485

[B33] Stevenson R. A. Sun S. Z. Hazlett N. Cant J. S. Barense M. D. Ferber S. (2016). Seeing the forest and the trees: default local processing in individuals with high autistic traits does not come at the expense of global attention. J. Autism Dev. Disord. 48, 1–15. 10.1007/s10803-016-2711-y 26861715

[B34] Stewart M. E. Watson J. Allcock A.-J. Yaqoob T. (2009). Autistic traits predict performance on the block design. Autism 13, 133–142. 10.1177/1362361308098515 19261684

[B35] Van Heer C. A. Crewther D. P. (2012). Orientation and spatial frequency selective surround suppression impairment in high autistic tendency. Front. Hum. Neurosci. 6:106. 10.3389/conf.fnhum.2012.208.0010622586383

[B36] van Loon A. M. Knapen T. Scholte H. S. John-Saaltink E. S. Donner T. H. Lamme V. A. (2013). GABA shapes the dynamics of bistable perception. Curr. Biol. 23, 823–827. 10.1016/j.cub.2013.03.067 23602476

[B37] Vattikuti S. Chow C. C. (2010). A computational model for cerebral cortical dysfunction in autism spectrum disorders. Biol. Psychiatry 67, 672–678. 10.1016/j.biopsych.2009.09.008 19880095 PMC3104404

[B38] Wetzels R. Wagenmakers E.-J. (2012). A default Bayesian hypothesis test for correlations and partial correlations. Psychon. Bull. Rev. 19, 1057–1064. 10.3758/s13423-012-0295-x 22798023 PMC3505519

[B39] Wheatstone C. (1838). XVIII. Contributions to the physiology of vision. —Part the first. on some remarkable, and hitherto unobserved, phenomena of binocular vision. Philos. Trans. Royal Soc. Lond. 128, 371–394. 10.1098/rstl.1838.0019

[B40] Wilson H. R. (2003). Computational evidence for a rivalry hierarchy in vision. Proc. Natl. Acad. Sci. U.S.A. 100, 14499–14503. 10.1073/pnas.2333622100 14612564 PMC283620

